# Cumulative Dose from Recurrent CT Scans: Exploring the DNA Damage Response in Human Non-Transformed Cells

**DOI:** 10.3390/ijms25137064

**Published:** 2024-06-27

**Authors:** Davide Valente, Maria Pia Gentileschi, Alessandro Valenti, Massimo Burgio, Silvia Soddu, Vicente Bruzzaniti, Antonino Guerrisi, Alessandra Verdina

**Affiliations:** 1 Unit of Cellular Networks and Molecular Therapeutic Targets, Department of Research and Advanced Technologies, IRCCS Regina Elena National Cancer Institute, 00144 Rome, Italy; davide.valente@cnr.it (D.V.); mariapia.gentileschi@ifo.it (M.P.G.); silvia.soddu@ifo.it (S.S.); 2 Institute of Molecular Biology and Pathology (IBPM), National Research Council (CNR), c/o Sapienza University, 00185 Rome, Italy; 3 Unit of Radiology and Diagnostic Imaging, Department of Clinical and Dermatological Research, IRCCS San Gallicano Dermatological Institute, 00144 Rome, Italy; alessandro.valenti@ifo.it (A.V.); massimo.burgio@ifo.it (M.B.);; 4 Unit of Medical Physics and Expert Systems, Department of Research and Advanced Technologies, IRCCS Regina Elena National Cancer Institute, 00144 Rome, Italy; vicente.bruzzaniti@ifo.it

**Keywords:** computed tomography, cumulative dose, DNA damage, γH2AX/53BP1 foci, focus size, radiation-induced foci (RIF), persistent-RIF

## Abstract

Recurrent computed tomography (CT) examination has become a common diagnostic procedure for several diseases and injuries. Though each singular CT scan exposes individuals at low doses of low linear energy transfer (LET) radiation, the cumulative dose received from recurrent CT scans poses an increasing concern for potential health risks. Here, we evaluated the biological effects of recurrent CT scans on the DNA damage response (DDR) in human fibroblasts and retinal pigment epithelial cells maintained in culture for five months and subjected to four CT scans, one every four weeks. DDR kinetics and eventual accumulation of persistent-radiation-induced foci (P-RIF) were assessed by combined immunofluorescence for γH2AX and 53BP1, i.e., γH2AX/53BP1 foci. We found that CT scan repetitions significantly increased both the number and size of γH2AX/53BP1 foci. In particular, after the third CT scan, we observed the appearance of giant foci that might result from the overlapping of individual small foci and that do not associate with irreversible growth arrest, as shown by DNA replication in the foci-carrying cells. Whether these giant foci represent coalescence of unrepaired DNA damage as reported following single exposition to high doses of high LET radiation is still unclear. However, morphologically, these giant foci resemble the recently described compartmentalization of damaged DNA that should facilitate the repair of DNA double-strand breaks but also increase the risk of chromosomal translocations. Overall, these results indicate that for a correct evaluation of the damage following recurrent CT examinations, it is necessary to consider the size and composition of the foci in addition to their number.

## 1. Introduction

Computed tomography (CT) is one of the most informative diagnostic imaging procedures that leads to accurate detection and management of several specific clinical situations, such as oncology, trauma, and chronic and cardiovascular diseases. Over the last decades, the use of CT has dramatically increased in many countries [1,2], and CT examination has become one of the main sources of medical exposure to ionizing radiations (IR) [3,4,5], especially for cancer patients, who are exposed to multiphasic CT procedures (multiple acquisitions of the same anatomical region) during the same session. This has led to increasing concerns on potential health risks particularly for individuals who are subjected to high number of CT examinations over a short period of time, such as patients with oncologic diseases. Other examples of individuals exposed to high number of CT scans, though with lower doses of IR, include patients with trauma or healthy individuals enrolled in periodically repeated health screening programs [6,7]. Indeed, the trend in total dose exposure have reversed in recent years [8], and non-multiphasic CT procedures expose patients to low doses of IR, even lower than 1 mSv. However, the cumulative effective dose received from recurrent multiphasic CT examinations, such as those required for oncologic patients, can be equal to or higher than 100 mSv [9,10,11,12]. This highlights the need to establish the biological consequences of these procedures to help define the appropriate criteria and guidelines to evaluate the risk/benefit ratio of each diagnostic procedure [13].

It is well known that IR induces DNA double-strand breaks (DSBs), one of the most serious injuries that can lead to cell death, replicative senescence, chromosomal aberrations, and eventually, neoplastic transformation [14,15,16,17,18]. Cells respond to the presence of DSBs by the DNA damage response (DDR), a sophisticated machinery that senses the damage and orchestrates the repair process [19,20,21,22]. Key steps in the DDR to DSBs is the phosphorylation of histone H2AX (γH2AX) at the sites of damage [23,24,25] and its recognition by the p53-binding protein 1 (53BP1) [26,27,28], which contributes to the formation of discrete radiation-induced foci (RIF). Detection of RIF by combined immunofluorescence (IF) of γH2AX and 53BP1 is considered a marker of DNA DSBs, and their quantification has been applied to examine the radiation effects in different conditions, including medical diagnostic radiation exposure [29,30,31,32,33]. In particular, after acute induction of DSBs, the number of γH2AX/53BP1 foci suddenly increases within 30 min–1 h to return to basal levels, upon DNA repair, in the following 24 h. It has been proposed that a few DSBs might not be repaired and persist for months, unless the cells die, and be responsible for both short- and long-term harmful effects, including the onset of cancer [34,35,36]. These unrepaired foci, also called “persistent repair foci”, have been shown to be larger (between 1.6 to 1.9 μm^2^), though qualitatively similar, than acutely formed or naturally occurring foci (0.3–0.4 μm^2^) [37,38]. Even larger γH2AX foci (between 5 to 17 μm^2^), rather than increased number of foci, have been detected upon cell exposure to high linear energy transfer (LET) radiations (e.g., alpha particles) [39]. In addition to these different-size RIF, in the past decade, large-scale movements of DSBs have been reported, including clustering, anchoring, and nuclear periphery movements. These movements yield to DSB compartmentalization into novel chromatin subdomains, the damaged or “D” topologically associating domains (TADs) that have been shown to facilitate DSB repairs [40]. However, this DSB-induced chromosome reorganization comes at the expense of genome integrity because it can increase the rate of chromosome translocations [41].

In this work, we investigated the biological effects of diagnostic doses of low-LET radiation produced by recurrent CT scans. To mimic the IR conditions applied to oncologic patients, immortalized human fibroblasts (HF) and retinal pigment epithelial (RPE-1) cells were maintained in culture for five months and subjected to four CT scans, one every four weeks. We observed that both the number and size of γH2AX/53BP1 foci significantly increased with CT scan repetitions and revealed the appearance of persistent giant foci morphologically similar to the recently described D-TAD.

## 2. Results

### 2.1. Recurrent CT Scans Increased the Number of Persistent γH2AX/53BP1 Foci

**Figure 1:**
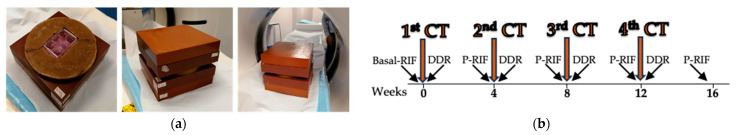
Irradiation of cultured cells by CT scan. (**a**) Left panel: the plate containing the cells was put inside a specially constructed 2 cm wax slab and placed on a 10 cm thick solid water phantom. Center panel: the phantom was covered with 2 slabs of solid water, each 5 cm thick. Right panel: CT was carried out so that the hemi-thickness of the plate was positioned at the isocenter of the tomograph. (**b**) Schematic representation of the experimental design. Basal RIF: count of the RIF present in basal condition, before any irradiation; P-RIF: count of persistent RIFs; DDR: count of RIFs at different time points for the evaluation of DDR kinetic.

To simulate the irradiation conditions of individuals during CT examinations, the plates containing either HF or RPE-1 cells were placed into a specifically constructed wax slab that was put inside a solid water phantom for dosimetry and quality-control in radiotherapy. The CT scans were performed so that the hemi-thickness of the plate was in the isocenter of the tomograph (Figure 1a) (see Section 4). At every CT scan, cells plated on coverslips were fixed before irradiation and at different time points post-irradiation (from 0.5 to 24 h) to evaluate the DDR kinetics and, in parallel, were maintained in culture for four weeks. After four weeks, the cells were analyzed for the presence of persistent-RIF (P-RIF). Then, the same experimental procedure was repeated four times, once every four weeks. Specifically, a new CT scan was performed with similar experimental conditions for a total of four CT scans administered to the same cell populations, as schematized in Figure 1b. We chose a four-week interval as an appropriate compromise to observe the long-term effect of multiple CT-scans considering that both HF and RPE-1 are able to repair acute damage in 24 h and that both had to be splitted once a week to maintain optimal culture conditions. 

**Figure 2:**
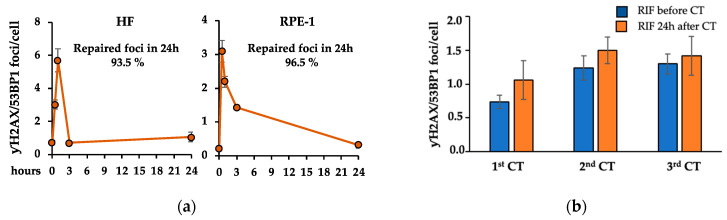
Evaluation of γH2AX/53BP1 foci. (**a**) DDR kinetic in the indicated cells were analyzed by IF detection of γH2AX/53BP1 foci at different time points (30 min, 1, 3, and 24 h). Each point represents the mean ± standard errors (SE) calculated on at least 50 cells per sample. The percentage of the repaired foci in 24 h is reported in the panel. (**b**) γH2AX/53BP1 foci in HF cells were measured before and 24 h after three recurrent CT scans. The baseline is represented by RIF before the first CT. Each point represents the mean ± SE calculated on at least 50 cells per sample. The differences between RIF at 24 h after CT and RIF before the following CT are not statistically significant.

The combined analysis of γH2AX/53BP1 foci by IF is considered a system of choice for the determination of DNA damage induced by low doses of IR [42,43]. Thus, we first analyzed the kinetic of DDR in HF and RPE-1 cells by detecting and counting the nuclear foci of co-localized γH2AX and 53BP1 at different time points (30 min, 1, 3, and 24 h) post-CT scan. As shown in Figure 2a, a significant phosphorylation of H2AX and the formation of γH2AX/53BP1 foci was readily observed in both cell lines at short times after IR exposure (1 h for HF and 30 min for RPE-1). Subsequently, a significant decrease was observed in both cell lines, earlier in HF compared to RPE-1. However, although the two cell lines showed a different speed of γH2AX dephosphorylation, the percent of repaired foci in 24 h was similar (i.e., repaired foci in 24 h in HF = 93.5% and repaired foci in 24 h in RPE-1 = 96.5%), indicating that cells from the two lines repair the DNA damage with a comparable efficiency. 

**Figure 3:**
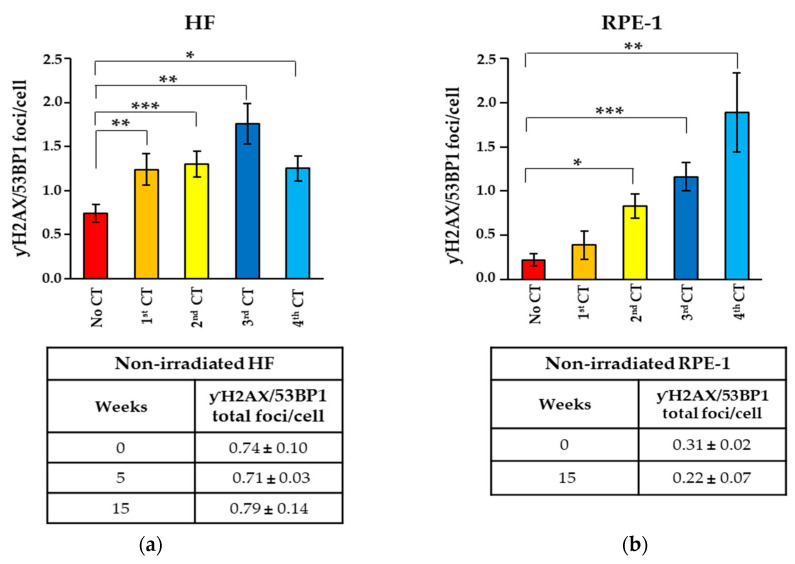
Evaluation of γH2AX/53BP1 foci in (**a**) HF and (**b**) RPE-1 cells undergoing recurrent CT scans or never being irradiated. The histograms show the levels of P-RIF foci measured after 1 month from each indicated CT scan and the underlying tables show the P-RIF at the indicated time in the parental, non-irradiated cells. Values represent the mean ± SE calculated on at least 50 cells per sample. The asterisks indicate the statistically significant differences as follows: * *p* < 0.05; ** *p* < 0.01; *** *p* < 0.001.

Next, we asked whether the small percentage of γH2AX/53BP1 foci, still present 24 h after the first CT scan, could be resolved during the following weeks. To this aim, we evaluated the γH2AX/53BP1 foci in HF cells before and 24 h after three recurrent CT scans and observed that the foci present after each CT scan were still maintained after four weeks, suggesting that they might not be resolved (Figure 2b). Thus, we measured the number of these persistent γH2AX/53BP1 foci in HF and RPE-1 cells four weeks after each CT for a total of four CT scans (Figure 1b). Parental cells maintained in parallel cultures but never subjected to CT scan were used as the control. We found a significant increase in the number of persistent γH2AX/53BP1 foci in both cell lines undergoing recurrent CT scans (Figure 3a), while no difference was observed in the non-irradiated cells cultured for similar periods of time (Figure 3b), indicating that the presence of persistent foci is independent from the time in culture. 

### 2.2. Recurrent CT Scans Increased the Size of Persistent γH2AX/53BP1 Foci

**Figure 4:**
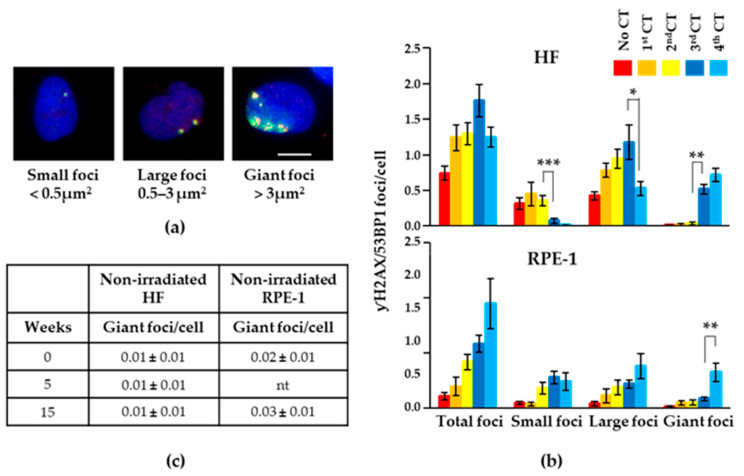
(**a**) Qualitative analysis of foci before the fourth CT in the HF cells. Cells were immuno-stained with anti-γH2AX (red) and anti-53BP1 (green) specific antibodies and DAPI (blue). Images were visualized by IF using a 60x objective. Representative pictures of foci with different sizes are reported. (**b**) Total, small, large, and giant γH2AX/53BP1 foci in HF and RPE-1 cells were measured after 1 month from each indicated CT scan. Values represent mean ± SE calculated on at least 50 cells per sample. Scale bar is 10 μm. Asterisk (*) indicates statistically significant differences as follows: * *p* < 0.05; ** *p* < 0.01; *** *p* < 0.001. (**c**) Giant foci in the parental cells that were never irradiated. Values represent the mean ± SE calculated on at least 50 cells per sample. nt: not tested.

During the consecutive counting of the γH2AX/53BP1 foci in the HF cells after the recurrent CT scans, we realized that, particularly after the third and fourth CT scans, the size of the foci increased significantly, and these foci appeared to be formed by the confluence of smaller foci, as shown in Figure 4a (right panel). Based on the size (i.e., focus areas), we defined three categories of foci, small foci: <0.5 μm^2^; large foci: 0.5–3 μm^2^; and giant foci: >3 μm^2^ (Figure 4a), and we counted their relative presence after every recurrent CT scan. We found that after recurrent CT scans, the number of small foci decreased while that of large and giant foci increased, with the latter representing the majority of the foci present after the fourth CT scan in the HF cells (Figure 4b upper panel). A very low number of giant foci was observed in the non-irradiated, parental cells, and this number did not change along the time in culture (Figure 4c). Comparable results were obtained with the RPE-1 cells (Figure 4b lower panel and Figure 4c).

### 2.3. Persistent Foci Did Not Induce Cell Cycle Arrest

**Figure 5:**
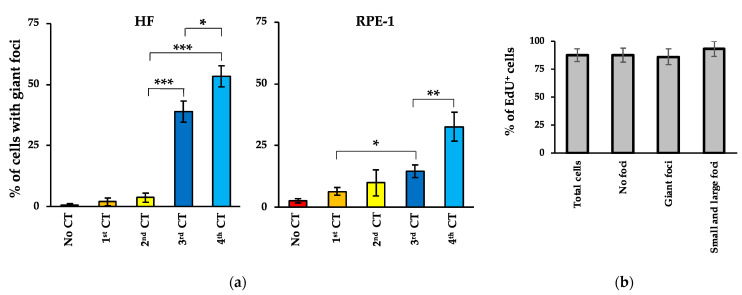
(**a**) The percentages of HF and RPE-1 cells showing giant foci were measured by IF after 1 month from each indicated CT. Values represent the mean ± SE calculated on at least 50 cells per sample. The asterisk (*) indicates statistically significant differences as follows: * *p* < 0.05; ** *p* < 0.01; *** *p* < 0.001. (**b**) DNA synthesis was measured in HF cells by EdU incorporation. The percentages of EdU-positive cells in the total cell population and in the cells with no foci, with giant foci, and with other size foci (large and small) is reported. Values represent mean ± SE calculated on at least 100 cells per sample.

It has been proposed that persistent foci are present in growth-arrested cells and associate with replicative senescence or cell death [34,44]. However, other authors have shown that persistent foci do not block cell proliferation [45,46]. Thus, we asked whether the persistent foci we observed after recurrent CT scans are associated with cell cycle arrest. First, we calculated the percentage of cells with giant foci and observed an increase after each irradiation cycle (Figure 5a), suggesting that these cells are not lost during cell passages in culture. Next, we directly evaluated DNA replication by measuring EdU incorporation into newly synthesized DNA. We found no significant difference in the percentage of EdU positivity among cells carrying giant foci, other foci (small and large), or no foci (Figure 5b). EdU-incorporation by DNA repair (i.e., low, spotted EdU-staining) was excluded from the evaluation. These data show that the persistent foci associated with recurrent CT scans did not induce cell cycle arrest.

### 2.4. Giant Foci Were Observed In Vivo in Peripheral Blood Mononuclear Cells

**Figure 6:**
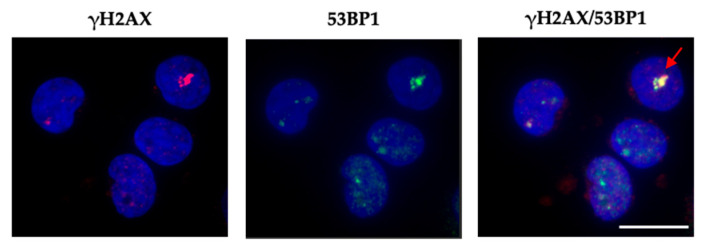
The figure shows representative images of γH2AX, 53BP1, and merging γH2AX/53BP1 foci in PBMCs from a healthy volunteer. Foci of different sizes including a giant focus (red arrow) are shown. The images were visualized by a fluorescence microscope using a 60x objective. Scale bar is 10 μm.

It has been proposed that at very low doses of X-rays, persistence of foci can be observed in primary human fibroblasts and murine tissues but not in peripheral blood lymphocytes, which instead show a rapid decline down to pre-exposure focus levels [47]. Thus, we asked whether the persistent foci we detected after recurrent CT scans in vitro on fibroblasts and epithelial cells can be observed in vivo in peripheral blood mononuclear cells (PBMCs). We isolated PBMCs from a healthy volunteer, who previously underwent periodic hip X-rays for orthopedic reasons, and performed IF for γH2AX/53BP1 foci as we did for HF and RPE-1 cells. Although present in very small numbers, foci of different sizes were observed in the PBMCs (Figure 6). In particular, we observed very large foci formed by the confluence of small foci, similar to those found in vitro, in cultured and irradiated adherent cells, indicating that they can also form in vivo in PBMCs.

## 3. Discussion

In the recent years, considerable concern has been linked to the increasing use of radiological exams, in particular CT scan, whose employment in diagnostic imaging has exponentially grown in several countries of the world [1,2,13]. Despite the low doses of IR employed, CT represents a risk factor for patients, as evidenced in large cohort studies [48]. Furthermore, several studies have raised the problem of the cumulative effective dose received by patients/individuals who undergo multiple radiological exams, highlighting the need to establish imaging appropriateness criteria and guidelines [9,10,11,12]. For a correct use of these procedures, the assessment of the risk/benefit ratio is essential and, consequently, the analysis of the damage resulting both from single and multiple CT exposures is necessary. 

In the present study, we exposed two human non-transformed cell models, HF and RPE-1, to four successive CT scans, executed four weeks apart one from the other, and analyzed the subsequent DNA damage by γH2AX/53BP1 IF detection of RIF. The CT-scan follow-up in patients is usually longer than 4 weeks. However, the interval in vitro was chosen, considering that cell cycle and proliferation in culture are faster. Since both RPE-1 and HF are able to repair acute damage in 24 h and both had to be splitted once a week to maintain optimal culture condition, we considered the 4 week interval as an appropriate compromise to observe the long-term effect of recurrent CT-scan. We found that cells from both models responded to CT-induced damage by activating the DDR mechanism that is able to repair about 95% of the damage, and this repair efficiency was not reduced after recurrent CT scans. However, a small percentage of foci escaped the repair, or were very slowly repaired, and they remained in the cells, accumulating after the subsequent CT-scans in the form of giant foci. The EdU-incorporation experiment indicates that cells with giant foci can proliferate at levels comparable to others, suggesting that cells with giant foci have the same fitness as the controls but, following repeated CT scans, they increase in number. However, since cells were splitted once a week, another possibility is that giant foci form as a result of cell division and do not persist as hypothesized by us. Time-lapse cellular models with cells expressing fluorescently tagged yH2AX and 53BP1 would be required to answer this important aspect. Overall, we found that recurrent CT scans increased both the number and the size of persistent γH2AX/53BP1 foci and that the presence of these foci is not associated with cell cycle arrest.

Investigating the nature and functions of these giant foci is undoubtedly interesting and will be explored in future work. However, the purpose of this manuscript is to argue that for a proper evaluation of DNA damage and associated risk, it is necessary to assess the size of the foci as well as their number.

Epidemiological studies have clearly shown an increased risk of developing leukemia and solid cancer in individuals exposed to high doses of IR (>0.1 Gy) [49]. In contrast, the risk of cancer following exposure to low doses of IR (<0.1 Gy) is still unclear, more difficult to quantify, and mainly extrapolated from studies of people accidentally exposed to very high radiation doses [50,51]. For example, by studying the atomic bomb survivors, the VII report of the Biologic Effects of Ionizing Radiation established a linear no-threshold model (LNT) to estimate the risk of radiation-related cancer in individuals exposed to low doses of IR [52]. However, while a large number of data support the LNT model at high doses, there are few pieces of evidence to sustain linear extrapolation at low doses. Indeed, the radiation hormesis hypothesis suggests that low-dose radiation can be beneficial to irradiated cells and organisms [53]. Furthermore, another significant effect related to low-dose radiation is the non-targeted effect, also referred to as the bystander effect [54]. Despite all these considerations, we cannot exclude that a small but real risk of developing cancer would exist even when the radiation doses are in the order of tens of mGy, i.e., one CT scan; however, definitive evidence on this issue is still lacking [55,56,57]. Indeed, while association between pediatric CT examination and cancer risk has been reported, other studies have failed to confirm this association [51,58,59,60,61,62]. 

As regards the damage caused by repeated exposures to low doses of IR, such as those produced during recurrent CT scans, little information is available. Recently, persistent DNA DSBs after repeated diagnostic CT scans have been described in the MCF10A breast epithelial cells [46]. In particular, the level of γH2AX and 53BP1 foci remained enhanced for up to six months after three recurrent CT scans, supporting the existence of a “memory effect” that might reflect a radiation-induced long-term response after repeated exposures of low-dose X-rays. Interestingly, as we also observed in our cells, no association with cell cycle arrest was observed in the MCF10A, excluding replicative senescence or proliferative exhaustion as the main mechanism behind RIF accumulation after recurrent CT scans [46]. 

During our study, we distinguished mainly three categories of foci based on the size, i.e., small, large, and giant foci. Previous studies showed that differences in the focus size are related to the type of radiation [37,38,39,63]. In particular, high-LET radiations, more effective in causing DNA damage compared to low-LET, induce larger foci due to clustered DSBs, a more difficult damage to repair compared to a simple DSB induced by low-LET radiations [64]. However, the different focus sizes induced by high and low LET radiations are evident only when high doses of radiation are used (i.e., 0.5 Gy) [39]. In our cells, we observed that the development of giant foci can be also induced by the accumulation of low doses of low-LET radiation (IR from recurrent CT scans). These giant foci, a few in number after the first CT scan, significantly increased after the third and fourth CT scans. Since the giant foci result from the overlapping of closely spaced small foci, counting the giant as single foci may lead to an underestimation of the damage if it is detected only based on the number of foci. This would explain the decrease in the number of total foci in HF four weeks after the fourth CT as a result of the increase in giant foci.

Even if the giant foci observed are induced by very low doses of low-LET radiations, for which the probability of having more than one DSB in a focus is considered negligible for single exposures, we cannot rule out whether giant foci may indicate a complex DNA damage that is very difficult to repair and that could potentially generate harmful effects [65]. Interestingly, at the morphological level, our giant foci resemble, both in size and γH2AX/53BP1 composition, the D-TADs recently identified in cells with inducible DSBs [41]. The D-TADs are chromatin compartments in which damaged DNA is assembled to regulate DDR. It has been proposed that, from one site, the D-TADs facilitate the repair of DNA DSBs; however, on the opposite site, they might also increase the risk of potentially oncogenic chromosome translocations [66]. It will be relevant to evaluate whether the giant foci induced by recurrent CT scans are mechanistically comparable to the experimentally induced D-TADs. At this point, whether the giant foci represent an accumulation of unrepaired DNA damage as reported following single exposition to high doses of high-LET radiation or reorganizing D-TADs is still unclear. However, their appearance after recurrent CT examinations strongly supports the need of taking into consideration the size of the foci, in addition to their number, when evaluating this type of DDR.

Overall, based on our results, we can conclude that, for the correct detection of the damage following repeated exposures to low doses of IR (CT scan) and for the evaluation of the risk/benefit ratio, it is necessary to consider the size and the composition of the foci in addition to their number. 

## 4. Materials and Methods

### 4.1. Cell Lines and Culture

Human hTERT-immortalized dermal fibroblasts (HF) [67] and retinal pigment epithelial (RPE-1) cells were maintained, respectively, in DMEM GlutaMAX and RPMI-1640 GlutaMAX media, both supplemented with 10% fetal bovine serum and 1% penicillin/streptomycin (all from Life Technologies, Carlsbad, CA, USA) and incubated in a humidified 5% CO_2_ atmosphere at 37 °C. For DDR evaluation, cells (6 × 10^4^) were seeded onto cover glasses previously coated with 0.1 mg/mL polylysine (Life Technologies, Carlsbad, CA, USA) and grown in six-well plates for three days to reach a 70% confluence before irradiation. Irradiated cells were maintained in culture for the following CT scans for a total of five months and four CT scans (see below). 

### 4.2. Irradiation (CT Scan)

Cells were exposed to irradiation by CT Scanner Philips model INCISIVE 128 slices. To simulate the irradiation conditions of individuals during CT examinations, the six-well plates containing the cells were put inside a specifically constructed 2 cm wax sheet/slab that was then placed on a 10 cm (2 slabs of 5 cm) thick phantom of solid water employed for absolute dosimetry and quality controls in radiotherapy. The whole was covered with two sheets of solid water of 5 cm each. The CT scans were performed so that the hemi-thickness of the wax sheet/slab was in the isocenter of the tomograph. The routine Abdomen Pelvis protocol was used with helical acquisition of four series of images corresponding, in the patient, to acquisitions of one basal image series and three series corresponding to arterial, portal-venous, and late phases. The typical scan parameters were 120 kV, 200 mAs, rotation time 0.5 s, slice thickness 2.5 mm with spacing between slices 1.5 mm, pitch 0.6, CTDI vol 65 mGy, dose length product (DLP) 2513.99 mGy·cm, and effective dose of 60.33 mSv associated with abdominal CT exam. CT scans were repeated on the same cell populations every four weeks for a total of four rounds. In more detail, for each CT scan, cells on cover glasses were subjected to CT scan and either fixed at different time points (i.e., 30 min, 1, 3, and 24 h) for DDR analysis or cultured for an additional four weeks until the next immunostaining or CT scan, while cells from the same population were fixed without undergoing CT scan. DDR was analyzed also four weeks after the fourth CT scan. Control untreated cells were maintained in culture for 15 weeks and transferred at room temperature during every irradiation time of the paralleled treated samples. Thus, both irradiated and control cells underwent the same changing in temperature. Control untreated cells were analyzed for DDR at the indicated time points.

### 4.3. Immunostaining/Immunofluorescence Assay

Cells cultured on cover glasses, irradiated or not, were fixed with 3.7% formaldehyde (Sigma-Aldrich, Burlington, MA, USA) in phosphate-buffered saline (PBS) (Life Technologies, Carlsbad, CA, USA) for 10 min and permeabilized using 0.25% Triton X-100 (Sigma-Aldrich, St. Louis, MO, USA) for 10 min, followed by two PBS washes and blocking for 1 h with 5% BSA (Sigma-Aldrich, St. Louis, MO, USA) in PBS. Next, cells on cover glasses were incubated overnight at 4 °C with a mixture of two antibodies, the mouse monoclonal anti phospho-histone H2AX-Ser139 antibody (Cell Signaling Technology, Danvers, MA, USA—Code 05636, diluted 1:500) and the rabbit polyclonal anti 53BP1 antibody (Bio-techne, Minneapolis, MN, USA) —Code NB 100-304, diluted 1:500). After three quick-washings and three five-minute washings in PBS, cells on cover glasses were incubated for 1 h at room temperature with a mixture of Goat Anti-Rabbit IgG diluted 1:800 (Alexa Fluor 488, Life Technologies, Carlsbad, CA, USA) and Goat Anti-Mouse IgG diluted 1:400 (Alexa Fluor 594, Life Technologies, Carlsbad, CA, USA). After six washings, as above, DNA counterstaining was obtained by adding 0.5 μg/mL of DAPI solution (Thermo Scientific, Waltham, MA, USA) for 10 min in the dark at room temperature. After an additional six washings, the cover glasses were mounted on glass slides using Vectashield mounting (DBA, Milano, Italy) and γH2AX/53BP1 foci visualized by an immunofluorescence microscope (Olympus BX53, Olympus Corporation of the Americas, Center Valley, PA, USA). 

### 4.4. Foci Analysis

The analysis of γH2AX/53BP1 foci was performed both manually and automatically by focus counting on acquired immunofluorescence images. Manual counting was blindly performed by counting at least 50 nuclei per image from at least five images per sample. Automated focus count and size was calculated by CellProfiler software (version 4.2.1) [68]. The focus size was measured as area in µm^2^ both manually, using ImageJ, and automatically, using the module “MeasureObjectSize” in CellProfiler software. Briefly, at least ten 60X images were analyzed for each condition. Colocalizing yH2AX/53BP1 foci were manually counted and, based on dimensions, they were categorized as small, large, and giant. To overcome operator bias, results were confirmed using an appropriately designed pipeline on Cellprofiler software.

The percentage of repaired foci was calculated as reported [69], according to the following formulas:

Number of induced foci = foci 30 min/foci 1 h − foci 0

Number of repaired foci = foci 30 min/foci 1 h − foci 24 h

Repaired foci in 24 h (%) = Number of repaired foci/Number of induced foci × 100

### 4.5. Cell Replication Assay

Cell replication was measured by 5-ethynyl-2′-deoxyuridine (EdU) incorporation into newly synthesized DNA and its recognition by azide dyes via a copper-mediated “click” reaction, using a Click-iT^®^ EdU Imaging Kit (Invitrogen, Waltham, MA, USA). Briefly, HF cells were incubated with 10 μM EdU for 24 h at 37 °C, fixed with 3.7% formaldehyde for 15 min, and treated to visualize EdU incorporation following the manufacturer’s instructions. 

### 4.6. Peripheral Blood Mononuclear Cells (PBMCs) Isolation

Blood samples (5 mL) were obtained from a healthy volunteer into ethylenediaminetetraacetic acid (EDTA) vacutainers, and PBMCs were immediately isolated by gradient centrifugation with Ficoll Paque PLUS (GE Healthcare, Chicago, IL, USA) at 2000 rpm for 20 min at room temperature. The layer containing the PBMCs was removed and washed twice with PBS. Then, PBMCs were counted, and 4 × 10^4^ cells were stuck on glass slide by cytocentrifugation (Shandon Cytospin 4, Thermo Electron Corporation, Waltham, MA, USA) 5 min at 600 rpm for γH2AX/53BP1 IF analysis. The study (RS1560/21) was conducted in accordance with recognized ethical guidelines (Declaration of Helsinki) and approved on June 22, 2021 by Comitato Etico Centrale IRCCS—Sezione IFO-Fondazione Bietti, Roma.

### 4.7. Statistical Analysis

Statistical analyses were performed using GraphPad Prism v.9 [70]. Differences between two groups were examined using the two-tailed Student’s *t*-test. *p* values < 0.05 were considered significant. Data are presented as bar plots with average foci number ± standard error (SE) of the mean per cell. Asterisks were used as follows: * *p* < 0.05; ** *p* < 0.01; *** *p* < 0.001.

## Data Availability

The data presented here are present in a deposed database.
